# Finishing Additively Manufactured Ti6Al4V Alloy with Low-Energy Electrical Discharges

**DOI:** 10.3390/ma16175861

**Published:** 2023-08-27

**Authors:** Dorota Oniszczuk-Świercz, Adrian Kopytowski, Rafał Nowicki, Rafał Świercz

**Affiliations:** Institute of Manufacturing Technology, Faculty of Mechanical and Industrial Technology, Warsaw University of Technology, Narbutta 85, 02-524 Warsaw, Poland; adrian.kopytowski@pw.edu.pl (A.K.); rafal.nowicki1@pw.edu.pl (R.N.); rafal.swiercz@pw.edu.pl (R.Ś.)

**Keywords:** Ti6Al4V, finishing surface, additive manufacturing, surface integrity, electrical discharge

## Abstract

Additive manufacturing has garnered significant interest in various industries due to its flexibility and capability to produce parts with complex shapes. However, issues related to surface quality, such as roughness and microstructural defects, necessitate the use of post-processing techniques to achieve the desired properties. Ti6Al4V alloy, produced additively, was finished using low-energy discharges, and the new surface integrity properties resulting from the induced heat energy were investigated. To further understand the influence of discharge energy on the formation of the new layer, roughness parameters and power spectral density were used to characterize the surface topography. SEM and EDS analyses were performed to examine the morphology and microstructural defects such as microcracks. The results indicate that the heat energy induced by the discharge improved the properties of the surface. SEM analysis revealed that the new layer was characterized by a reduction in defects such as unmelted particles, the balling effect, and microcracks. At the lowest investigated discharge energy of *E* = 0.21 mJ, surface roughness, *Sa*, was reduced by about 69%, which is equal to about 2 μm, accompanied by a significant decrease in microcracks. EDS analysis indicated that the diffusion of copper and zinc from the electrode to the top surface was related to the discharge energy. Furthermore, prediction models of the influence of wire electrical discharge polishing parameters, including discharge energy, wire speed, and time interval, on the surface roughness and material removal rate (*MRR*) were developed using the response surface methodology.

## 1. Introduction

Metal additive manufacturing (MAM) technologies are increasingly being integrated into various industries. These new manufacturing capabilities are reshaping the approach to designing parts and assembling devices, enabling the creation of structures that were impossible to produce using conventional machining technologies [1]. Among the many MAM technologies, selective laser melting (SLM) is one of the most frequently employed under production conditions. SLM allows for the rapid fabrication of geometrically complex parts directly from CAD models using hard-to-machine materials such as Ti6Al4V titanium alloy. The layer-by-layer production of parts satisfies engineers’ requirements related to low weight (through lattice-based geometries) and strength. This potentially facilitates the production of parts from titanium alloy for various applications, including in the space, automotive, energy, and medical industries.

In the SLM process, a laser beam induces the local melting of the powder and substrate. The complex physical phenomena associated with the impact of thermal energy induced by the laser beam—conduction, radiation, convection, heat absorption by powder particles and the substrate, powder melting, the solidification of molten metal, and shrinkage—present significant challenges to determining the optimal SLM process parameters [2]. The high temperature gradient, rapid cooling, and thermal cycling in successive scan passes generate tensile stresses [3,4]. The surface integrity of SLM-produced Ti6Al4V alloy is characterized by defects such as high roughness, microcracks, the balling effect, or unmelted particles, which necessitate removal via additional post-processing techniques [5,6,7,8]. Obtaining the required surface properties involves selecting the appropriate finishing treatment, such as machining [9,10], abrasion [11], hot isostatic pressing [12], heat treatment [13,14], laser polishing [15,16], shot peening [17], or electrochemical polishing [18]. The choice of post-processing technology affects surface roughness, fatigue life, residual stress, microstructure, and microhardness [19,20].

One of the new research directions for improving the surface properties of MAM parts is the utilization of low-energy electrical discharge interactions. An example of this technology is wire electrical discharge polishing. As a result of electrical discharge interactions between the working electrode in the form of a thin wire and the machined surface, excess material is removed. During the electrical discharge, a narrow plasma channel is formed through which the current flows, causing the local melting and vaporization of the material. Micro-craters are created at the discharge points, forming a new surface topography. A local high heat flux leads to changes in the material’s structure [21,22,23,24,25]. Numerous variables that influence the stability of electrical discharges, such as discharge current intensity, discharge time, discharge voltage, electrode tension, dielectric pressure, and electrode speed, make it difficult to identify the impact of individual variables on the material removal process and the quality of the machined surface [26,27,28,29,30,31].

Several studies have focused on modeling and optimizing the process [32,33,34]. Relatively few works have analyzed the influence of wire electrical discharge polishing (WEDP) process parameters on the surface integrity of additively manufactured parts [35,36,37,38]. Abhilash et al. [39] reported that the use of WEDP enhanced the dimensional accuracy of finished parts, particularly reducing radial and corner errors by up to 80%. Jithinrajal et al. [40] highlighted that enhanced dimensional accuracy is related to the selection of an appropriate number of finishing passes. The proposed solutions enable a reduction in shape errors, such as flatness or inclined surfaces, and cylindricity. Notably, these errors were reported to be reduced by about 70% compared to those in the original model. Research conducted by Thasleem et al. [41] indicates that electrical discharges can be used to alloy SLM parts with the elemental composition of the electrode. Electrical discharge alloying enhances hardness and wear resistance because it applies a new layer of material containing carbide (SiC) and oxide (Al_2_O_3_).

Existing research indicates that wire electrical discharge polishing can significantly improve surface properties as well as dimensional and shape accuracy. However, the effects of low-energy discharges on the functional properties of additively manufactured Ti6Al4V surfaces, particularly in relation to topography, morphology, and the material removal rate, have not been sufficiently described. Therefore, this study presents the results of an experimental investigation on the influence of discharge energy on surface quality.

This research was focused on establishing a relationship between low-energy electrical discharges on the surface properties of SLM Ti6Al4V alloy. The heat flux induced by discharges leads to the melting and evaporation of the material and electrode, generating a newly formed layer. To characterize the properties of the new topography, the function of discharge energy surface roughness parameters along with power spectral density analysis were used. SEM and EDS analyses were performed to examine morphology and microstructural defects, such as microcracks.

Furthermore, an analysis of the influence of the technological parameters of wire electrical discharge polishing (including wire speed and time interval), which affect the stability and sustainability of the process, was conducted, focusing on their impact on surface roughness (*Sa*) and the material removal rate (*MRR*).

## 2. Materials and Methods

The main focus of this work was to examine the effects of low-energy electrical discharges on the surface quality of additively manufactured Ti6Al4V alloy. Selective laser melting (SLM) technology was used to prepare samples from titanium powder using an EOSINT M100 machine (EOS GmbH, Krailling, Germany). The parameters of the SLM process used are presented in Table 1. In the final stage of the manufacturing process, the samples were heat-treated for 1 h in a vacuum furnace at a temperature of 850 °C.

The additively manufactured Ti6Al4V samples with low energy discharges were polished using a Charmilles 440 wire EDM machine (GF, Bienne, Switzerland). During the experiment, the samples were connected to the positive pole of the current, and the working electrode in the form of a thin wire had negative polarity.

Based on the analysis of current and voltage waveforms during the finishing operation, a range of electrical parameter variability, within which stable electrical discharges were observed, was determined. These parameters include discharge current intensity, discharge voltage, pulse duration, and the time interval between discharges. It should be noted that the machine tool generator used does not allow for the independent adjustment of parameters such as discharge current and pulse duration. As the pulse duration increases, the discharge current also increases. These relationships were analyzed and the discharge voltage in the energy of the electric discharge was calculated in accordance with Equation (1):

(1) $$E=\int_{0}^{t_{on}}{U_{c}\left(t\right)\cdot I}_{c}\left(t\right)dt\;(mJ),$$

where *U_c_* is the discharge voltage (V), *I_c_* is the discharge current (A), and *t_on_* is the discharge time (μs).

Detailed information on the measuring circuit developed for the measurement and analysis of voltage and electric current waveforms during electro-erosion cutting treatment was presented in previous research [42].

The finishing conditions used in the experimental tests of the wire electrical discharge polishing (WEDP) process are presented in Table 2.

In the second stage of the research, the main focus was on determining the influence of the WEDP process parameters discharge energy (*E*), wire speed (W), and time interval (*t_off_*) on surface roughness (*Sa*) and the material removal rate (*MRR*). Experimental studies were conducted using Hartley’s experimental design, with 5 levels and 3 parameters. Table 3 shows the ranges of input parameters for individual levels of the planned experiment.

The results of the experimental studies were used to build prediction models with the response surface methodology. The material removal rate was calculated in accordance with Equation (2):

(2) $$MRR=\frac{l\times h}{∆t}\left[\frac{{mm}^{2}}{min}\right]$$

where *l* is the cutting length, *h* is the sample height, and ∆t is the manufacturing time.

**Figure 1:**
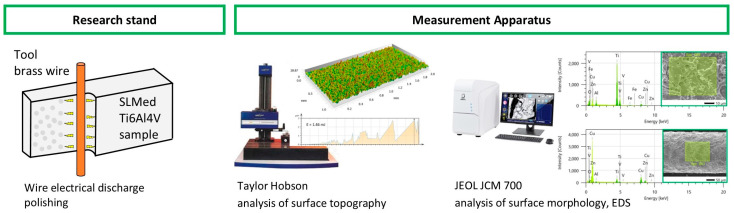
Scheme of experimental setup.

Surface integrity analysis was conducted on high-end equipment. The topographic properties were measured with a Talysurf series 2 scanning profilometer (Taylor Hobson, Leicester, UK). The samples were measured on a 1 mm × 2 mm area. SEM and EDS analyses of the surface were performed on JEOL JCM-7000 NeoScope (Tokyo, Japan). The scheme of the methodology for measuring surface integrity is presented in Figure 1.

## 3. Results and Discussion

### 3.1. Surface Morphology Properties

**Figure 2:**
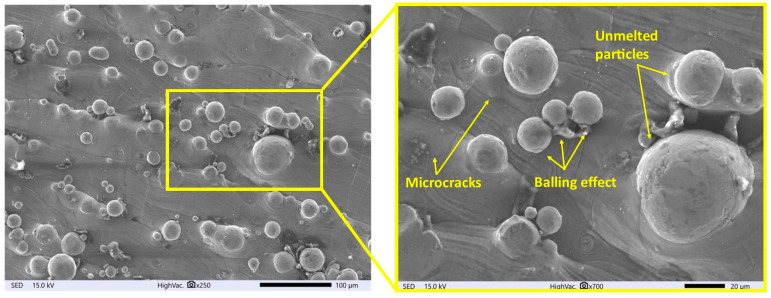
Surface of SLM Ti6Al4V sample.

Surface integrity properties significantly affect the tribological properties and fatigue strength of parts [43]. An analysis of the surface morphology of titanium samples manufactured with SLM indicates the presence of undesirable features, such as high surface roughness, microcracks, and unmelted material particles (Figure 2). During the SLM process, thermal processes resulting from laser impact cause a significant temperature gradient within the material. The magnitude of tensile residual stress increases toward the outer surfaces along the build direction [44]. As a result of shrinkage, tensile residual stresses are generated, leading to the formation of microcracks. The occurrence of the balling effect is an important factor that affects the quality and densification of SLM-produced parts. The accumulation of spherical particles is a result of surface tension, which prevents the even distribution of liquid metal on the surface [45].

**Figure 3:**
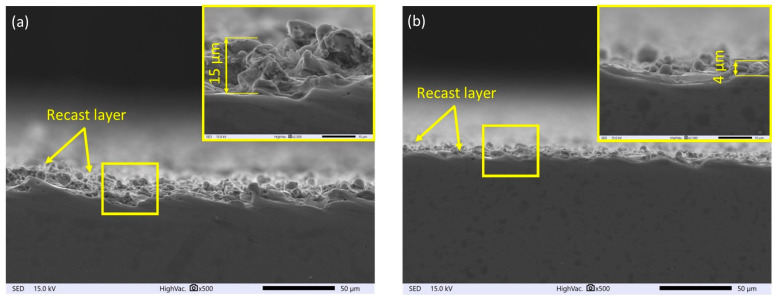
Surface morphology of Ti6Al4V after finishing with electrical discharge energy; (**a**) *E* = 1.46 mJ, and (**b**) *E* = 0.21 mJ.

One of the main goals of the proposed WEDP post-processing technology is to improve the quality of the obtained surfaces. New properties of the surface layer are formed as a result of the impact of the heat flux from the plasma channel generated during the electric discharge between the working electrode and the surface of the workpiece. According to the models of Gaussian heat flux propagation inside the plasma channel, rapid thermal processes cause the local melting and vaporization of both the workpiece and the working electrode [46]. A gas bubble filled with vaporized liquid metal forms around the plasma channel. At the moment of implosive closure of the discharge crater and gas bubble, the processing products are ejected into the inter-electrode gap [47]. Cross-sectional analysis of the SEM images of SLM Ti6Al4V after finishing indicates that the amount of re-solidified material undergoes significant changes based on the amount of discharge energy (Figure 3). The portion of material not removed during the erosion process re-solidifies on the machined surface, creating a new material structure. A decrease in discharge leads to a reduction in the recast layer’s thickness. For the highest discharge energy, *E* = 1.46 mJ, the recast layer’s thickness reached about 15 μm (Figure 3a), and for the lowest discharge energy, the recast layer’s thickness was about 4 μm (Figure 3b).

**Figure 4:**
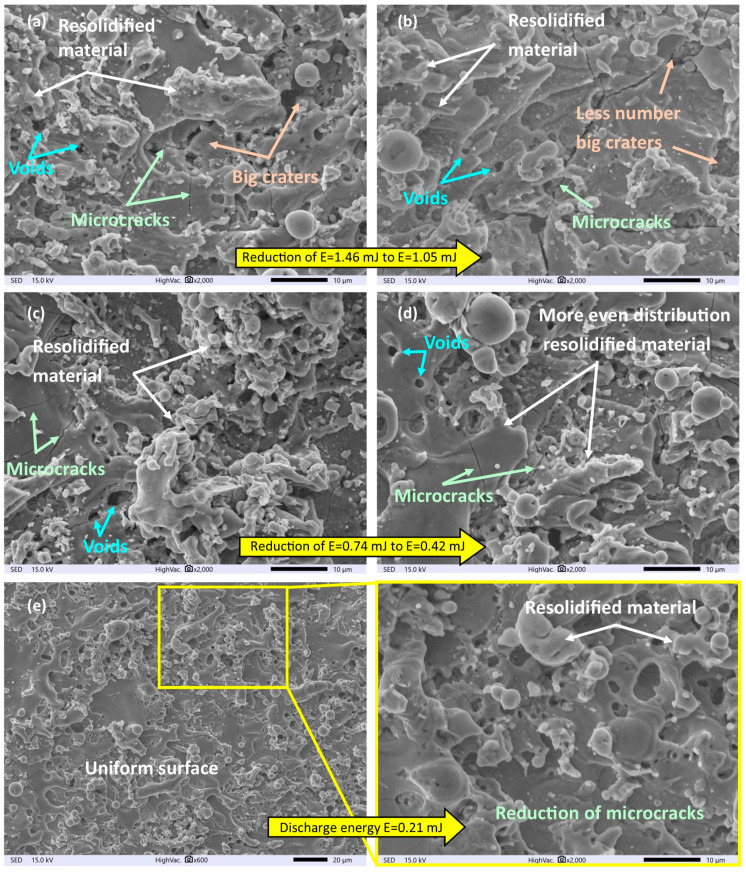
Surface morphology of Ti6Al4V after finishing with electrical discharge energy; (**a**) *E* = 1.46 mJ, (**b**) *E* = 1.04 mJ, (**c**) *E* = 0.74 mJ, (**d**) *E* = 0.42 mJ, and (**e**) *E* = 0.21 mJ.

The analysis of SEM images (Figure 4) indicates that the surface morphology significantly changes as a result of the impact of electric discharges. The new layer is characterized by the presence of re-solidified material, the amount of which directly depends on the amount of discharge energy. Decreasing the discharge energy leads to a reduction in the amount of eroded material in a single pulse, resulting in smaller crater diameters (Figure 4a,b). This also leads to a decrease in the volume of material that has not been evaporated and re-solidified on the surface. Reducing the discharge energy leads to the leveling of the surface and a more uniform distribution of the melted layer. However, when energy was in the range of 1.4 to 0.4 mJ, microstructural defects in the form of microcracks and voids were observed (Figure 4a–d). The presence of microcracks adversely affects the fatigue strength of the material and can contribute to intergranular corrosion [48].

The source of microcrack formation is the interaction between the heat flux inside the plasma channel and the high temperature gradient in the material. The part of the material that has not evaporated as a result of the electric discharge re-solidifies on the surface of a much cooler core. The generated tensile stress, after exceeding the permissible strength, causes the formation of microcracks [49]. Another undesirable feature on the surface is the presence of voids, which indicate the uneven melting of the material in the SLM process. The influence of electric discharges on the surface leads to the vaporization and then solidification of a specific volume of material. If the treated surface has pores or discontinuities in the form of unmelted/not fully melted powder, the process of material melting and heat conduction may take a different course.

In areas where pores are present, they will be “exposed” and the melted material will not distribute evenly on the surface. In the case of material discontinuities due to unmelted powder particles, the transport of thermal energy flux may be uneven, leading to the non-uniform local melting and evaporation of the material and its re-solidification. The analysis of the morphology of the sample processed with the lowest discharge energy indicates surface leveling with a significant reduction in the occurrence of microcracks (Figure 4e).

**Figure 5:**
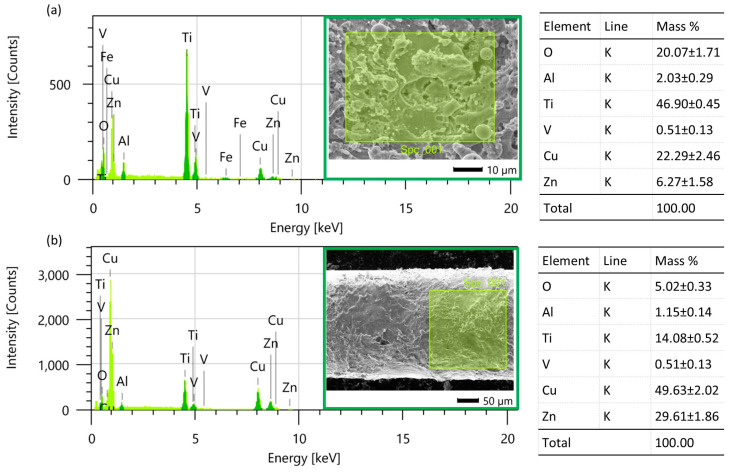
EDS element’s mass composition after electrical discharges with *E* = 1.46 mJ; (**a**) finished surface of Ti6Al4V, and (**b**) surface of wire electrode.

**Figure 6:**
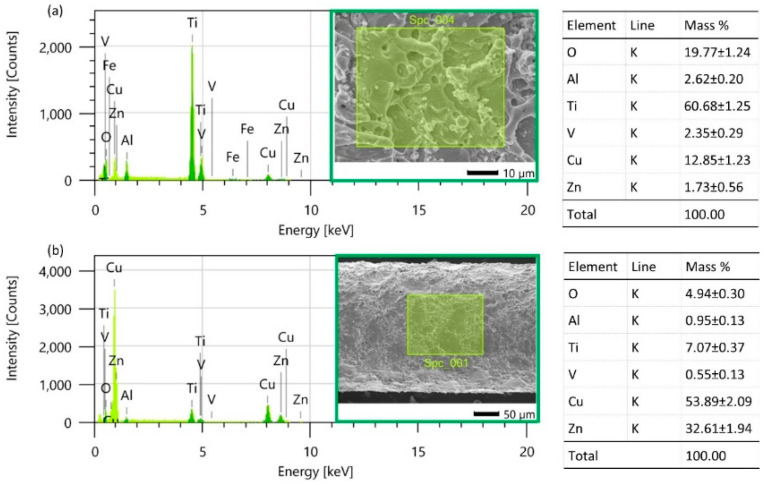
EDS element mass composition after electrical discharges with *E* = 0.21 mJ; (**a**) finished surface of Ti6Al4V, and (**b**) surface of wire electrode.

The process of electric discharge, during the which melting and evaporation of the working electrode and the workpiece occur, causes the formation of vapors of the molten materials and the diffusion of particles from the working electrode to the workpiece and vice versa. The EDS studies indicate that for the highest tested discharge energy, copper and zinc (components of the brass electrode) were diffused by 22% and 6%, respectively (Figure 5a). On the surface of the working electrode, titanium, aluminum, and vanadium were diffused by 14%, 1%, and 0.5%, respectively (Figure 5b). Reducing the discharge energy leads to a decrease in the heat delivered to the workpiece and the duration of its interaction. This results in a reduction in the amount of melted/evaporated material and limits the diffusion of elements. With the lowest electric discharge energy, copper and zinc were diffused by 13% and 2%, respectively (Figure 6a). On the surface of the working electrode, titanium, aluminum, and vanadium were diffused by 7%, 1%, and 0.5%, respectively (Figure 6b).

The EDS studies reveal a direct correlation between electric discharge energy and elemental changes on the machined surface. During electric discharge, the heat flux within the plasma channel causes both the workpiece and the working electrode to melt and evaporate. This results in the formation of a gas bubble filled with vapors of the molten material surrounding the plasma channel. As the electric discharge energy intensifies, the volume of material melted and evaporated from the electrode increases, leading to the increased diffusion of elements.

### 3.2. Analysis of Surface Topography Properties

The surface topography of titanium produced via the SLM process is characterized by many unfavorable features, such as significant differences in the height of the peaks due to the presence of unmelted/partially melted powder particles and the balling effect. To describe the properties of the manufactured surface, 3D roughness parameters (according to ISO 25178) were used, including the arithmetic mean of the deviation from the mean (*Sa*), the height of roughness (Sz), the maximum peak height of the surface (Sp), and the maximum valley depth of the surface (Sv). Parameters Sp and Sv are essential for evaluating surface properties in terms of the contact characteristics of mating surfaces. The value of Sp, which represents the highest peak height, is particularly relevant for sliding applications. Conversely, Sv provides insights into the fluid retention properties of the surface.

**Figure 7:**
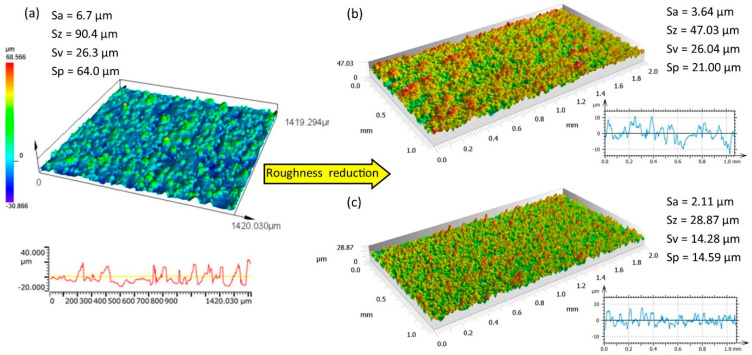
Surface topography of (**a**) SLM Ti6Al4V samples; SLM samples after finishing with discharge energies (**b**) *E* = 1.46 mJ and (**c**) *E* = 0.21 mJ.

The analysis of surface roughness parameters obtained after the SLM process indicates high values for the roughness amplitude. The arithmetic mean of the deviation from the mean was *Sa* = 6.7 μm, the roughness height was Sz = 90 μm, the peak height of the surface was Sp = 64 μm, and the deepest valley of the surface was Sv = 26.3 μm (Figure 7a). The above values are unacceptable in most cases regarding base or contact surfaces. The use of low-energy electrical discharges creates a new surface with different properties. A new surface topography is formed by the overlapping of individual electric discharge traces. For the proposed finishing technology, the craters have an asymmetric shape with an uneven distribution of the remelted layer [50]. During the discharge, the electrode moves linearly with the feed in the XY plane and in the *z* axis at a specified wire speed, causing the discharge to take place during the complex movement of the working electrode. Furthermore, electric discharge takes place in the dielectric (deionized water) supplied to the gap. After the end of electric discharge, the dielectric rapidly cools the molten metal and washes away the products of processing. A comparative analysis of the surface topography shows that, as a result of the impact of electric discharges, it is possible to significantly reduce the roughness height, and the resulting surface is characterized by an even distribution of roughness (Figure 7b,c). The experimental results are presented in Table 4.

**Figure 8:**
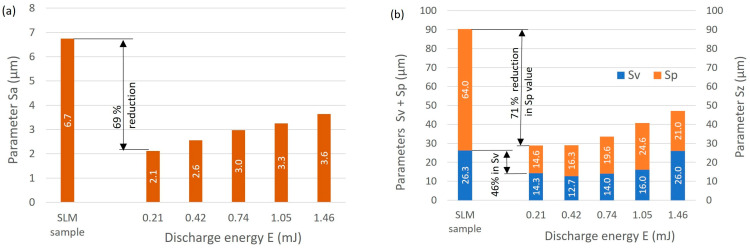
Improvement in the roughness parameters after WEDP; (**a**) *Sa*, (**b**) Sv and Sp.

Depending on the electrical discharge energy applied, the maximum reduction in roughness height was about 70%. With the lowest discharge energy, *E* = 0.21 mJ, *Sa* was reduced by 69% (Figure 8a) and Sp was reduced by 71% (Figure 8b). These relationships indicate that reducing the electric discharge energy leads to a decrease in the volume of melted and vaporized material, which results in the formation of craters with smaller depth. This leads to a decrease in peak height, Sp, and valley depth, Sv. The roughness height, Sz with discharge energy of 0.21 and 0.4 mJ was similar at about 29 μm, with craters at the lowest energy characterized by fewer peaks in relation to valleys. This results from the volume of removed and re-solidified material in a single discharge. With a discharge energy of 0.41 mJ, there was an increase in the amount of heat delivered to the material, leading to increased crater depth, but also an increase in the amount of melted material that was not removed during the erosion process and re-solidified on the surface of valleys (partially filling them) and the peaks. With the lowest energy, *E* = 0.21 mJ, the volume of material that re-solidified on the surface was the smallest and was more evenly distributed. Similar trends were reported by Boban et al. [51].

**Figure 9:**
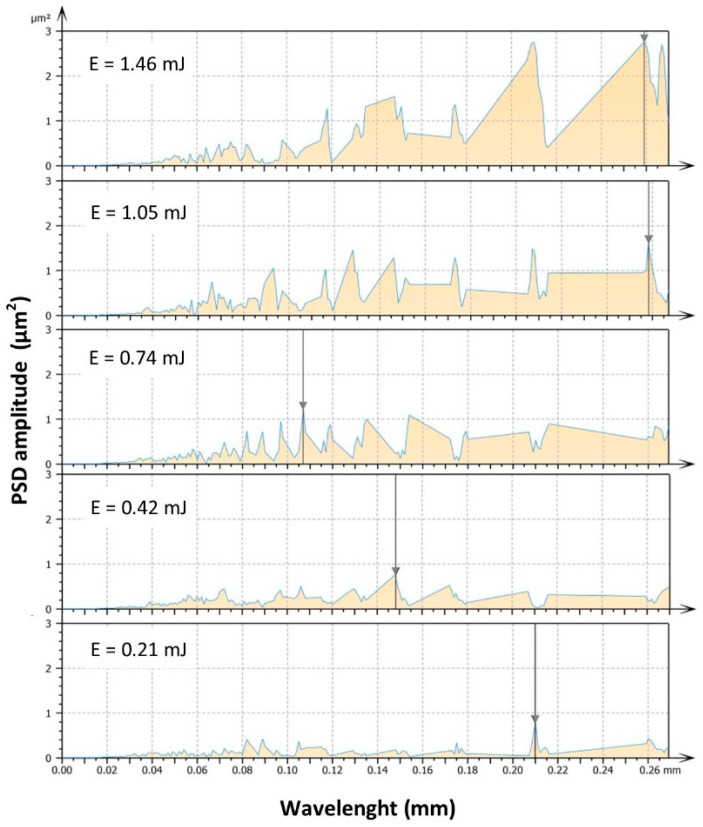
Power spectrum density of finished surface of Ti6Al4V for different discharge energy values.

The similar values obtained for parameters Sv and Sp, and the resulting Sz for discharge energies of *E* = 0.21 and 0.4 mJ, indicate that it is necessary to analyze the additional parameters describing topographic features in order to verify the correctness of the dependencies. One of the new methods for assessing surface conditions is power spectral density (PSD), which reveals spatial frequencies on the surface. Analyzing the amplitude–frequency relationship for a specific wavelength allows us to assess the impact of electrical discharge energy on surface characteristics described by height and wavelength [52]. The presented PSD graphs for the finishing treatment of titanium with low-energy discharge indicate a significant correlation between electrical discharge energy and the amplitude and length of waves (Figure 9). The spectra consist of different numbers of peaks with sizes ranging from 0.82 to 2.82 μm^2^ and wavelengths within the range of 0.107 to 0.259 mm.

**Figure 10:**
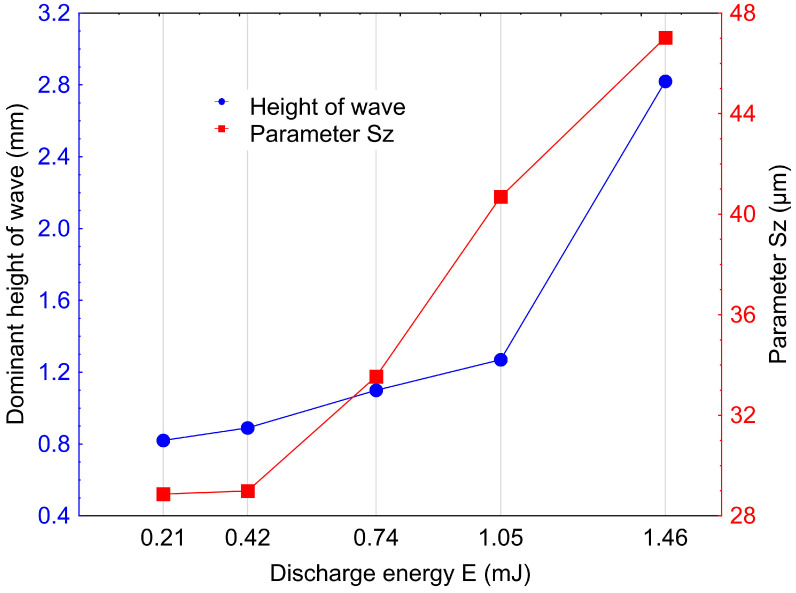
Dominant wave height and Sz as functions of discharge energy.

With the highest discharge energy, there are four main dominant peaks. Reducing the discharge energy leads to both an increase in the number of dominant peaks and a significant reduction in their height. The observed highest frequency with a relatively small height of individual waves for the lowest discharge energy indicates the presence of roughness with a low height and small spacing between peaks. As the energy of individual discharges increases, the frequency decreases, which indicates an increase in surface roughness. The relationship between the height of the dominant wave and the roughness parameter Sz shown in Figure 10 indicates a similar trend. Sz describes the amplitude of roughness, while PSD relates to wave frequency and height. Analyzing the surface topography characteristics based on parameters such as PSD and Sz leads to similar conclusions regarding the impact of discharge energy on surface features. The influence of a potential measurement error is negligibly small.

### 3.3. Prediction Models of Process

The experimental studies were aimed at determining and describing the influence of discharge energy on the quality of the Ti6Al4V surface prepared via SLM technology. Discharge energy, wire speed, and time interval were determined as the input parameters of the WEDP process. According to the research results, the amount of electric discharge energy affects the process of melting and evaporation of the material and determines both the morphology and topography of the surface. The wire speed affects the stability of the process. During electrical discharges, material is removed from both the workpiece and the working electrode. The craters formed on the surface of the working electrode cause a change in its mechanical properties (tensile strength), which can lead to breakage when a certain tension force is applied to the electrode. With the proper speed of the working electrode, the wear is negligibly small and does not affect the stability of the process. The final studied process parameter, the time interval, is responsible for stabilizing the machining conditions in the gap between discharges and indirectly influences the frequency of electrical discharges. Experimental studies were conducted in accordance with a methodology of a design of experiments for a five-level, three-parameter plan. The independent variables were defined as follows: discharge energy, *E* (mJ), wire speed, WS (m/min), and time interval, *t_off_* (µs). Eighteen tests with Ti6Al4V SLM finishing using WEDP were conducted. Table 5 presents the processing parameters along with the results of the experimental tests. Roughness, *Sa*, was in the range of about 2.11 to 3.64 μm, while the material removal rate (*MRR*) ranged from about 23 to 49.5 mm^2^/min.

The results of the experimental tests were used to develop predictive models. These models describe the impact of independent variables—electric discharge energy, wire speed, and time interval between discharges—on *Sa* and *MRR*. The prediction function is given by

*F* = *f* (*E*,*t_off_*,*WS*) ± *ε* (3)

where *F* is the response, which can be either *Sa* or *MRR*; *f* represents the regression function; *E* is the electric discharge energy; *t_off_* is the time interval; *WS* is the wire speed (these are the independent parameters); and *ε* denotes the experimental error.

To analyze the significance of terms in individual regression equations, an analysis of variance (ANOVA) was conducted at a 95% confidence level. The ANOVA results for the *Sa* and *MRR* models after removing non-significant terms are presented in Table 6 and Table 7. For each model, the coefficient of determination, R^2^, and the adjusted coefficient of determination, R^2^-adj, were calculated.

The analysis of the individual components of the regression equations in the roughness models shows electric discharge energy has the most significant impact on model variance; specifically, the values are 87.75% for the *Sa* model and 93.76% for the *MRR* model. While other terms and their interactions are statistically significant (at the 95% confidence level), their effect on the models is less pronounced.

Table 6 and Table 7 show that the calculated F-values are significantly greater than one, underscoring the statistical significance of the model at the chosen 95% confidence level. The R^2^ values were high, at over 97% for the *Sa* model and over 99% for the *MRR* model. These statistical values show that the response function aligns closely with the experimental results.

Using the ANOVA findings, response functions detailing the effects of the tested independent variables (electric discharge energy, wire speed, and time interval) on surface roughness (*Sa*) and *MRR* were developed. The refined response function for *Sa* and *MRR* is represented by the following polynomial functions:

*Sa* = 1.85 + 2.06 *E* − 0.56 *E*^2^ + 0.02 *WS* − 0.03 *t_off_*^2^ (μm) (4)

*MRR* = −8.81 + 63.38 *E* − 21.02 *E*^2^ + 0.54 *WS* + 2.79 *t_off_* (mm^2^/min) (5)

**Figure 11:**
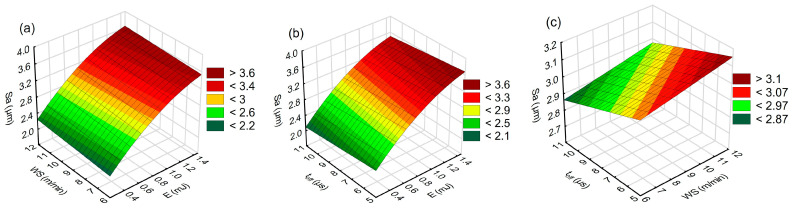
Response plots for the model of roughness (*Sa*); (**a**) *t_off_* = 8 µs, (**b**) *WS* = 9 m/min, and (**c**) *E* = 0.7 mJ.

**Figure 12:**
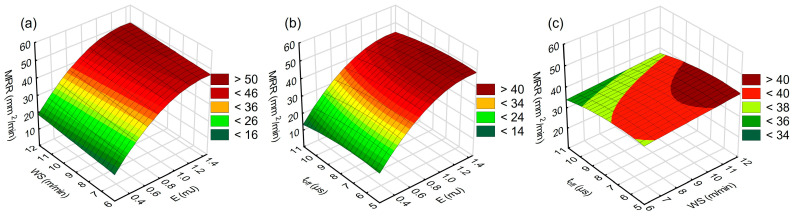
Response plots for the model of *MRR*; (**a**) *t_off_* = 8 µs, (**b**) *WS* = 9 m/min, and (**c**) *E* = 0.7 mJ.

Figure 11 and Figure 12 show graphical interpretations of the established relationships between discharge energy, wire speed, time interval, surface roughness and *MRR*, respectively.

The relationships shown in Figure 11 and Figure 12 indicate the impact of electric discharge energy on roughness (*Sa*) and *MRR*. Both functions, reflecting the roughness value and the material removal rate, exhibit similar trends. They are directly influenced by the volume of material removed in a single pulse. As the discharge energy increases, so does the heat flux density within the plasma channel. This density causes an increase in the volume of material that melts and vaporizes, leading to an expansion of both the depth and diameter of the crater. Consequently, the amplitude of roughness increases, influencing the *Sa* value and raising the *MRR*.

The graphs also indicate that the wire speed and the time interval between discharges are not decisive parameters affecting the material removal process (Figure 11a,b). Under constant energy, increasing the time interval causes a slight decrease in roughness (Figure 11c). Extending the time interval between pulses increases the evacuation time of treatment products from the gap, which positively influences the stabilization of treatment conditions. This results in a reduced probability of electric discharge being initiated on treatment products, which could lead to process instability and increased roughness [53]. The dependence also indicates that with the increased wire speed of the working electrode under constant discharge energy, the *Sa* roughness slightly increases.

An analysis of the influence of the time interval and wire speed on the *MRR* (Figure 12c) indicates that reducing the time interval to 7–8 μs increases the *MRR*. Reducing the time interval leads to an increased frequency of electrical discharges, which results in increased *MRR*. The observed local optimum of *t_off_* occurs because when the time interval is too short, the efficiency of removal of treatment products from the gap decreases, leading to unstable discharges and a consequent decrease in *MRR*.

In the analyzed range of variability, *WS* had a slight impact on *MRR* and *Sa*. However, the choice of speed is important for a sustainable material economy. Using a high wire speed does not significantly improve the stability of the process, but it significantly affects the use of natural resources and the cost.

## 4. Conclusions

This paper focused on the effect of low-energy electrical discharges on the surface integrity properties of additively manufactured Ti6Al4V. The experiments were divided into two stages. In the first stage, the impact of electrical discharge energy on surface integrity was characterized through SEM and EDS analysis and topography, including roughness amplitude and power spectrum density characteristics. In the second stage, the influence of WEDP parameters including discharge energy, wire speed, and time interval on roughness (*Sa*) and *MRR* was established. The research allowed us to arrive at the following conclusions:

1. Low-energy electrical discharges created a new surface layer with improved properties compared to those of the surface resulting from selective laser melting (SLM). Typical defects of the SLM surface, such as high roughness, unmelted particles, the balling effect, and the occurrence of microcracks, were minimized.
2. The value of discharge energy directly influenced the surface integrity properties, characterized by surface morphology, the thickness and composition of the recast layer, amplitude, and specific topographic parameters. Decreasing the discharge energy led to improved surface integrity properties.
3. Decreasing the discharge energy led to a reduction in locally induced thermal energy and the gradient of temperature delivered to the workpiece. The amount of material removed and re-solidified on the surface decreased. The maximum improvement of surface layer parameters was achieved with a discharge energy value of E = 0.21 mJ. The surface was uniform, with a significant reduction in the occurrence of microcracks. The recast layer thickness was about 4 μm. The reduction in roughness parameters *Sa*, Sp, and Sv was about 70%, 45% and 46%, respectively.
4. The relationship between power spectrum density and discharge energy indicates a significant correlation between electrical discharge energy and the amplitude and length of waves. Reducing the discharge energy led to an increase in the number of dominant peaks and a significant reduction in their height. As the energy of individual discharges increased, the frequency decreased, indicating an increase in surface roughness.
5. The relationships between discharge energy, roughness (*Sa*), and *MRR* exhibited similar trends. As the discharge energy increased, the heat transferred to the workpiece also increased, leading to a growth in the volume of material that melted and vaporized. Consequently, the roughness amplitude increased, influencing the value of *Sa* and increasing the *MRR*.
6. Wire speed and the time interval between discharges influenced the stability of the process, but these were not the main parameters affecting the material removal process. With an increase in the wire speed of the working electrode under constant discharge energy, roughness slightly increased. Reducing the time interval (*t_off_*) led to an increase in the frequency of electrical discharges, resulting in an increase in *MRR*. A local optimum was observed within the time range of 7–8 μs. However, reducing *t_off_* beyond this point did not lead to improved *MRR*.
7. Wire speed had a minor impact on both *MRR* and *Sa*. Nonetheless, wire speed is crucial for sustainable material usage. A decrease in wire speed does not significantly alter the stability of the process, but it does markedly affect the consumption of natural resources and the cost of the electrode.
8. Further studies should be carried out on the formation of the recast layer and its properties, including changes in elemental composition, XPS, nano-hardness, thickness, and residual stresses, as a function of discharge energy. Another important area of future research is to characterize the influence of the proposed finishing technology of additively manufactured parts on fatigue life.

## Figures and Tables

**Table 1:** SLM machining conditions.

Build Direction	Z Direction on Plate
Dimension of build sample	5 mm × 5 mm × 80 mm
Build chamber	oxygen below 0.1%
Titanium powder particles	20 μm to 40 μm
Laser power	250 W
Scanning speed	400 mm/s
Layer thickness	30 μm
Hatch spacing	100 μm

**Table 2:** WEDP machining conditions.

Electrode	Brass Wire, Diameter 0.25 mm
Workpiece material	Ti6Al4V
Discharge energy, *E*	0.21–1.46 mJ
Open voltage, *U*_0_	220 V
Dielectric	Deionized water

**Table 3:** Experimental design matrix.

Level	Parameter		
	Discharge Energy *E* (mJ)	Wire Speed (m/min)	Time Interval *t_off_* (µm)
−1.68	0.21	6	5
−1	0.42	7	6
0	0.74	9	8
1	1.04	11	10
1.68	1.46	12	11

**Table 4:** Roughness parameters after finishing WEDM.

Roughness Parameters	SLM Sample	Discharge Energy (mJ)				
		0.21	0.42	0.74	1.05	1.46
*Sa* (µm)	6.74	2.11	2.55	2.97	3.25	3.64
Sz (µm)	90.3	28.9	28.99	33.54	40.69	47.03
Sv (µm)	26.3	14.28	12.66	13.98	16.04	26.04
Sp (µm)	64.0	14.59	16.33	19.56	24.64	21

**Table 5:** Results of experimental studies.

Exp. no.	WEDP Input			Experimental Results	
	Discharge Energy *E* (mJ)	Wire Speed WS (m/min)	Time Interval *t_off_* (μs)	*Sa* (μm)	*MRR* (mm^2^/min)
1	0.42	7	6	2.55	27.4
2	0.42	7	10	2.57	23.4
3	0.42	11	6	2.59	28.8
4	0.42	11	10	2.58	25.1
5	1.05	7	6	3.37	43.1
6	1.05	7	10	3.13	41.8
7	1.05	11	6	3.41	46.7
8	1.05	11	10	3.25	44.3
9	0.21	9	8	2.11	16.9
10	1.46	9	8	3.64	49.5
11	0.74	6	8	2.96	38.5
12	0.74	12	8	3.13	41.1
13	0.74	9	5	3.18	39.9
14	0.74	9	11	2.94	37.1
15	0.74	9	8	2.99	39.2
16	0.74	9	8	2.96	39.4
17	0.74	9	8	2.93	39.6
18	0.74	9	8	2.97	39.5

**Table 6:** ANOVA results for *Sa*.

Source	Sum of Squares	Degrees of Freedom	Mean Square	F-Value	Prob > *f*	Contribution%
Model	2.33	4	0.58	8.98		
*E*	2.18	1	2.18	438.25	<0.0001	93.76
*E* ^2^	0.08	1	0.08	16.67	0.002	3.57
*WS*	0.02	1	0.02	9.46	0.024	0.74
*t_off_*	0.04	1	0.04	9.02	0.010	1.93
Error	0.06	13	0.06			
Total SS	2.39	17	R^2^ = 0.97		R^2^-adj = 0.96	

**Table 7:** ANOVA results for *MRR*.

Source	Sum of Squares	Degrees of Freedom	Mean Square	F-Value	Prob > *f*	Contribution%
Model	1423.46	5	284.69	17.84		
*E*	1249.09	1	1249.09	939.29	<0.0001	87.75
*E* ^2^	133.23	1	133.23	100.19	<0.0001	9.36
*WS*	15.01	1	15.01	11.29	0.005	1.05
*t_off_*	19.47	1	19.47	14.64	0.002	1.37
*t_off_* ^2^	6.65	1	6.65	5.00	0.045	0.47
Error	15.96	12	1.33			
Total SS	1302.46	17	R^2^ = 0.99		R^2^-adj = 0.98	

## Data Availability

Data will be made available on request.
